# Tactile stimulation in very preterm infants and their needs of non-invasive respiratory support

**DOI:** 10.3389/fped.2022.1041898

**Published:** 2022-11-18

**Authors:** Maxi Kaufmann, Barbara Seipolt, Mario Rüdiger, Lars Mense

**Affiliations:** ^1^ Department of Pediatrics, Division of Neonatology and Pediatric Intensive Care Medicine, TU Dresden, Medical Faculty Carl Gustav Carus, Dresden, Germany; ^2^ Saxony Center for Feto-Neonatal Health, TU Dresden, Medical Faculty Carl Gustav Carus, Dresden, Germany

**Keywords:** neonatal resuscitation, tactile stimulation, delivery room management, very preterm infants, respiratory support

## Abstract

**Aim:**

Despite the lack of evidence, current resuscitation guidelines recommend tactile stimulation in apneic infants within the first minutes of life. The aim was to investigate whether timing, duration or intensity of tactile stimulation influences the extent of non-invasive respiratory support in extremely preterm infants during neonatal resuscitation.

**Methods:**

In an observational study, we analyzed 47 video recordings and physiological parameters during postnatal transition in preterm infants below 32^0/7^ weeks of gestational age. Infants were divided into three groups according to the intensity of respiratory support.

**Results:**

All infants were stimulated at least once during neonatal resuscitation regardless of their respiratory support. Only 51% got stimulated within the first minute. Rubbing the feet was the preferred stimulation method and was followed by rubbing or touching the chest. Almost all very preterm infants were exposed to stimulation and manipulation most of the time within their first 15 min of life. Tactile stimulation lasted significantly longer but stimulation at multiple body areas started later in infants receiving prolonged non-invasive respiratory support.

**Conclusion:**

This observational study demonstrated that stimulation of very preterm infants is a commonly used and easy applicable method to stimulate spontaneous breathing during neonatal resuscitation. The concomitant physical stimulation of different body parts and therefore larger surface areas might be beneficial.

## Impact

**What is already known about this topic?**

• Tactile stimulation of infants at need of respiratory support is recommended in current resuscitation guidelines, independent of gestational age.
• Approaches of tactile stimulation vary widely within and between different centers.
• Extremely preterm infants receive tactile stimulation less common and shorter than more mature infants.
• Repetitive stimulation and a larger stimulated surface area might be beneficial.

**What this paper adds?**

• Tactile stimulation is routinely used in preterm infants at need of respiratory support independent of gestational age and an easy-to use method to stimulate spontaneous breathing.
• Concomitant stimulation at different body parts might be beneficial.

**How this study might affect research, practice or policy?**

• Prospective RCTs should focus on early concomitant tactile stimulation.
• Potentially negative stress caused by prolonged tactile stimulation should be further evaluated.

## Introduction

Premature birth is usually associated with disturbed respiration, making artificial ventilation a common procedure during postnatal stabilization (1). Mechanical ventilation however, is considered to damage the immature lung (2) and impairs brainstem development, causing long term respiratory sequelae and impaired neurodevelopmental outcome at preschool age (3). In recent years, postnatal management in the delivery room has therefore changed from routine intubation and ventilation to non-invasive respiratory support using continuous positive airway pressure (CPAP) for spontaneously breathing infants. If spontaneous breathing is insufficient, non-invasive ventilation with either initial inflations (INFL) or intermittent positive pressure (iPPV) is administered (4).

The majority of extremely preterm infants, e.g., born <28^0/7^ weeks of gestational age (GA), present with long apneic periods after birth (5). Since apnea is associated with an adducted larynx (6, 7), noninvasive ventilation is often impeded in these infants (8). Thus, efforts are focused on initiating spontaneous breathing. Although no standardized, evidence-based approach exists, current resuscitation guidelines suggest tactile stimulation (TS) as an integral part of supporting neonatal transition, besides drying and warming (4, 9). Despite of animal studies showing that TS has an influence on spontaneous breathing efforts in newborns (10, 11), there is still a lack of clinical evidence (12, 13) regarding optimal duration or method of tactile stimulation.

Although stimulation is regularly used to support neonatal transition, there is a huge variability between and within different centers, depending on experts' opinions and longtime experience (14–19). Some evidence exists that repetitive stimulation after birth improves oxygenation in preterm infants (20). However, caregivers stimulate extremely immature infants less frequently than more mature infants (15, 17). The routine placement inside a plastic wrap and concerns regarding skin integrity might hinder TS in the extremely preterm neonate even further.

In our institution, delivery room management is focused on preventing invasive ventilation in extremely preterm infants. The majority of infants is admitted to the neonatal intensive care unit (NICU) on CPAP. According to our experience and previous data (21), some infants achieve immediate respiratory stability (IRS) without any non-invasive ventilation, whereas others do need either or both forms of non-invasive ventilation (iPPV or INFL) to achieve respiratory stability. The time until respiratory stability is achieved varies in the latter group, but until now it remains unclear whether different clinical courses are caused by differences in tactile stimulation.

The present observational study was performed to test the hypothesis that more aggressive tactile stimulation is associated with a shorter period of non-invasive ventilation and an early respiratory stability (ERS), whereas less aggressive stimulation causes late respiratory stability (LRS), e.g., a longer need of non-invasive ventilation. The time of onset, duration and place of tactile stimulation were compared in very preterm infants with either ERS or LRS. Results were compared with control infants with immediate respiratory stability (IRS).

## Methods

### Population and data collection

All available video recordings of the postnatal transition of very preterm infants with a gestational age < 32^0/7^ weeks, born between January 2018 and October 2020, were eligible for analysis. Video recordings were excluded for insufficient quality (recording started after placing the newborn on the resuscitation unit, impaired field of view, camera failure) or if newborns had congenital malformations affecting the resuscitation. The video recordings show the resuscitation table and bedside monitoring and were analyzed as described previously (22).

Infants who received non-invasive ventilation were either grouped into ERS or LRS, if duration of non-invasive ventilation was below or above the median duration of non-invasive ventilation in the entire group. Control infants (IRS) did not receive any non-invasive ventilation.

### Routine delivery management of very preterm infants

Very preterm infants with a gestational age < 32^0/7^ weeks or birth weight < 1,500 g are placed in a plastic wrap under the radiant heater of a Giraffe® Shuttle. The caregiving team consists of a neonatologist, a pediatric resident and a NICU nurse. The delivery room (DR) management adheres to local guidelines in accordance with the current international recommendations at the time (23). All very preterm infants are treated with mask CPAP with a positive end-expiratory pressure of 8–10 cmH_2_O immediately after arrival on the resuscitation cot. The initial FiO_2_ is set at 0.3 and adapted according to the preductal oxygen saturation, aiming to achieve published reference values (24). Tactile stimulation and non-invasive ventilation are administered if considered necessary by the individual caregiver. Surfactant is administered in the delivery room according to local guidelines after the infant is considered respiratory stable on CPAP, typically after about 15 min of life (21). After placing a peripheral venous line and starting glucose infusion, skin-to-skin care is performed routinely in the delivery room at an average age of 50 min for at least 20 min.

### Video recordings and analysis

At our level III perinatal center video recordings of the neonatal resuscitation is recommended for all infants born < 32^0/7^ or birth weight < 1,500 g. One video camera is placed besides the resuscitation cot to record the infant and the hands of the caregivers. A second camera is installed in front of the pulse oximeter (Masimo Radical, Irvine, California). The ManyCam software is used to record a picture-in-picture video. For this study, videos were analyzed using the Interact® software (Interact® - Mangold International GmbH, Arnstorf, Germany), a video-based observation software with qualitative and quantitative data analysis capability, as described in more detail previously (22). All videos were reviewed by one investigator (MK) following a standardized protocol. Interrater variability was tested by independent review of 5 randomly selected videos by two investigators (MK, LM).

### Quantification of tactile stimulation

We captured data on onset, frequency and duration of each stimulation episode, and the respective method of tactile stimulation (feet, leg, back or chest). Subsequently, the overall time of stimulation was calculated. If there was no clear assignment to the stimulation method, for instance rubbing over the whole back or the side of the body it was defined as “various stimulation”. A new stimulation episode was defined as a gap of at least 3 s in between two stimulations or a change in the stimulation method. Stimulations of different areas at the same time are recorded as concomitant stimulations. Since very preterm newborns are placed inside a plastic wrap, drying is not performed routinely. Positioning the baby and placement of the pulse oximetry probe were not defined as TS.

### Quantification of respiratory support

All kinds of non-invasive respiratory support were analyzed regarding start and duration of the intervention. An episode of iPPV ceased once 10 s elapsed between two inspiratory positive pressure applications. “Respiratory stability” was defined as the time 10 s after the last application of iPPV or INFL.

### Statistical analysis

Results are presented as median (IQR) and as numbers (%). Infants were grouped by timepoint of respiratory stability and comparisons were performed using the Mann-Whitney *U* test for non-parametric variables and the *X*^2^-test for categorial variables. A *p*-value < 0.05 was deemed statistically significant (Supplementary Table S1). Data was analyzed using IBM SPSS Statistic version 28 (IBM Software, New York).

### Ethics

Video recordings were implemented for quality assurance and auditing at our level III perinatal center. It is part of the routine patient care and use of video recordings for retrospective research was approved by the Ethics Committee of the Technical University Dresden (EK 555122019).

## Results

### Population

90 video recordings were eligible during January 2018 and October 2020. 51/90 video recordings were complete and of adequate quality. Videos of 4 infants had to be excluded due to antenatal comorbidities affecting resuscitation significantly (pleural effusions, severe anemia, fetal lung hypoplasia). Therefore, the video recordings of 47 infants were included in our analysis. Characteristics of video graphed infants are depicted in Table 1. The median GA was 26^4/7^ [24^6/7^;28^6/7^] weeks with a median birth weight of 745 g [645 g;940 g]. Most of them were born by cesarean section and had received antenatal corticosteroids. Comparing the three different groups, no significant differences were observed except for the 5 min-APGAR Score (*p* = 0.010).

**Table 1:** Baseline characteristics.

	overall infants (*N* = 47)	IRS (*N* = 12)	ERS (*N* = 18)	LRS (*N* = 17)
weeks of GA^a^	26^4/7^ (24^6/7^;28^6/7^)	27^1/7^ (26^3/7^;28^5/7^)	26^5/7^ (24^3/7^;28^6/7^)	26^1/7^ (24^3/7^;28^1/7^)
Sex, male (%)	25 (53.2)	7 (58.3)	8 (44.4)	10 (58.8)
Birth weight, g^a^	745 (645;940)	743 (701;1,224)	738 (675;880)	805 (570;970)
antenatal corticosteroids (%)	45 (95.7)	12 (100)	16 (88.9)	17 (100)
cesarean delivery (%)	45 (95.7)	12 (100)	18 (100)	15 (88.2)
5 min APGAR score^a^	8 (6;8)	8 (8;9)	8 (6;9)	7 (6;7)

^a^ Median (IQR); LRS, late respiratory stability: resp. stability > 320 s; ERS, early respiratory stability: resp. stability ≤ 320 s; IRS, immediate respiratory stability: no need of intermittent positive pressure ventilation or initial inflations.

### Respiratory support

**Figure 1:**
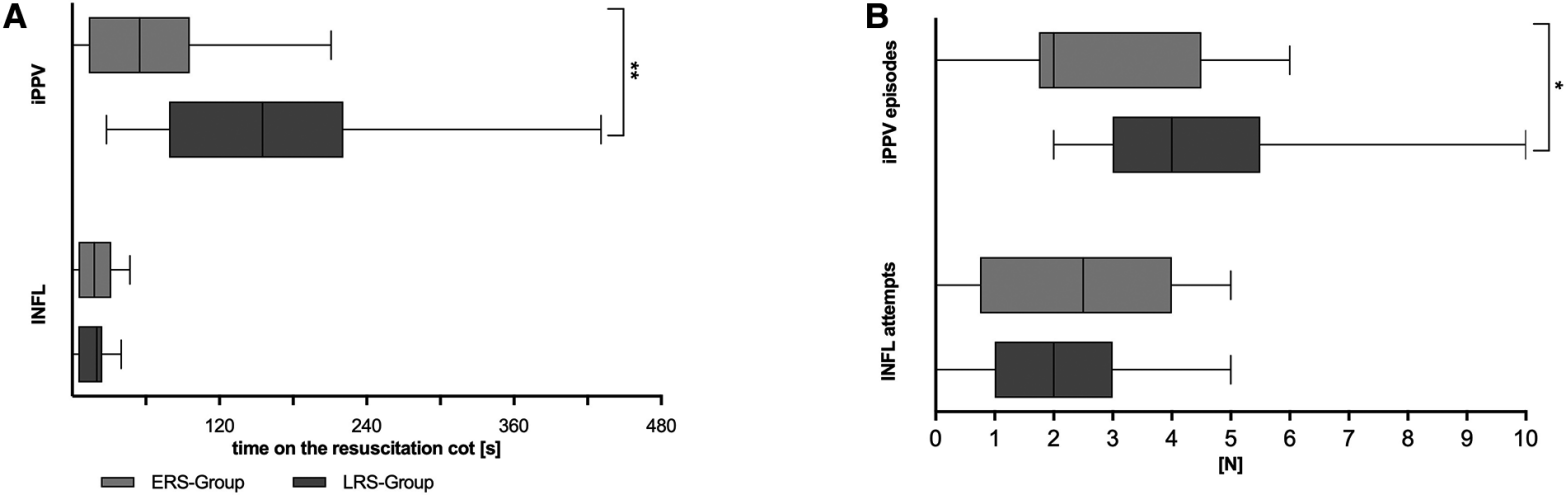
Respiratory support. Overall time used for intermittent positive pressure ventilation (iPPV) or initial inflations (INFL) (**A**) as well as iPPV-episodes and INFL-attempts (**B**) are shown for the LRS, late respiratory stability group and ERS, early respiratory stability group during the first 15 min of postnatal support. *p*-values - **p = 0.004, *p = 0.027.

In infants receiving non-invasive ventilation, respiratory stability was achieved at 320 s [230 s;427 s]. For subsequent analyses, infants were divided into ERS and LRS groups if non-invasive ventilation lasted more or less than 320 s, respectively (Figures 1A,B). These infants, except one in the ERS group which received INFL only, received at least one episode of iPPV. 76% and 78% of the infants in the LRS and ERS group, respectively received INFL at least once. Infants in the LRS group received 2 [1;3] and in the ERS group 2.5 [0.75;4] attempts of INFL. Infants in the LRS group received iPPV longer and in more episodes than those in the ERS group (Figures 1A,B).

### Timing of tactile stimulation

All infants received TS during the first 15 min of postnatal support at least once. TS was started in most of the infants (83%) within the first minute of postnatal support. Median time of initiation of TS was 57 s [26 s;105 s], without any highly significant difference between the three groups (LRS 39 s [20.5 s;100 s], ERS 56 s [28.25 s;100 s], IRS 83.5 s [57 s;122.5 s]).

**Figure 2:**
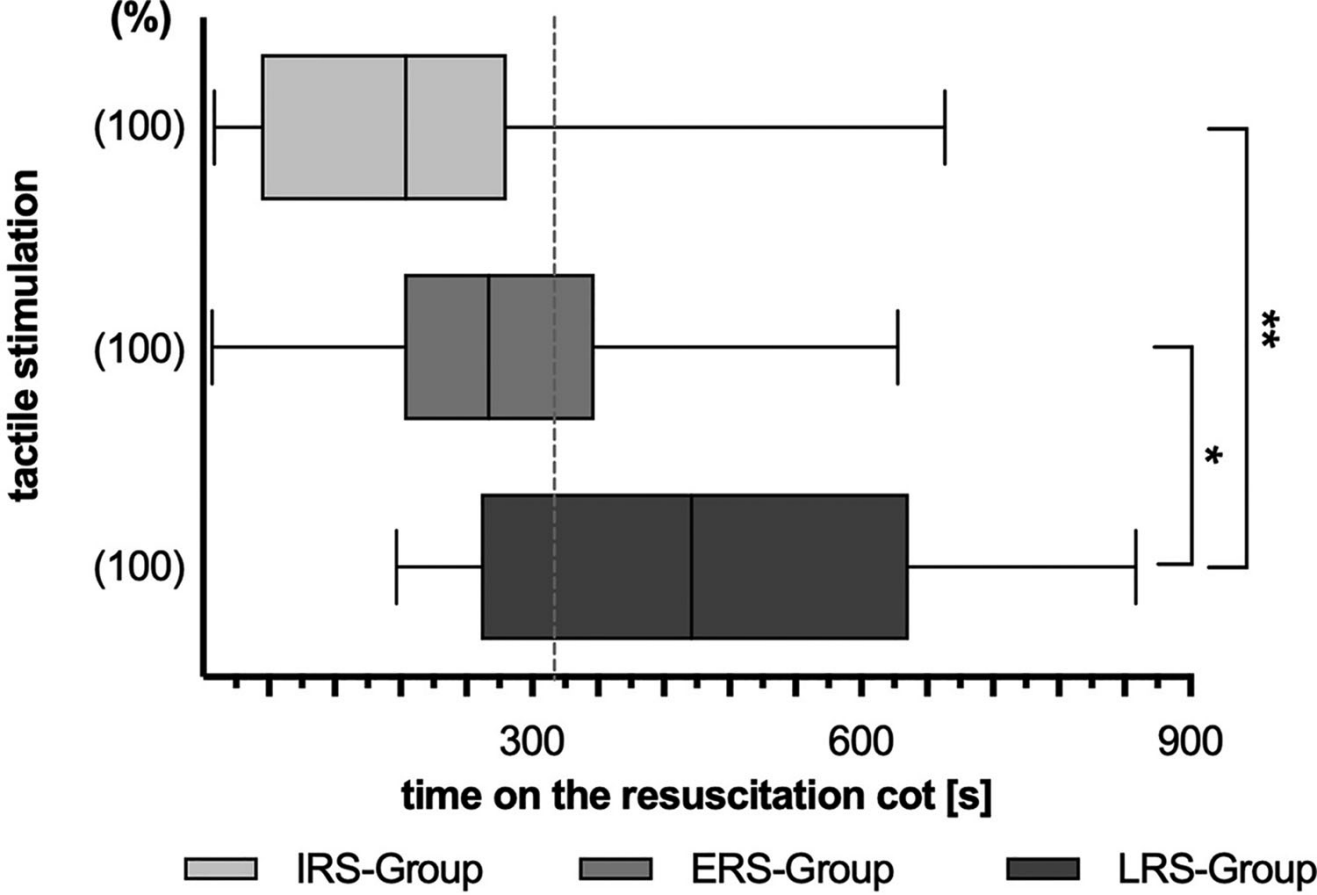
Overall duration of active tactile stimulation (TS) during the first 15 min of postnatal support. Three subgroups are depicted: LRS, late respiratory stability group; ERS, early respiratory stability group; IRS, immediately respiratory stability group, no need of iPPV or INFL within the first 15 min of postnatal support. (%) – proportion of infants within the particular group; *p*-values - **p = 0.003, *p = 0.010.

Among all infants, TS lasted for 278 s [193 s;447 s]. Overall duration of TS was significantly longer in the LRS group [445 s (253 s;643 s)] when compared with the ERS or IRS group respectively (260 s [183.25 s;356.75 s] and 184.5 s [52.5 s;276.75 s]), however, there was a wide range within the groups (Figure 2).

Except in two infants, TS was discontinued for more than 3 s at a median time of 221 s [113.5 s;376 s]. However, TS was discontinued significantly earlier in the IRS group [102.5 s (72.25 s;118.5 s)] when compared with the LRS or ERS group respectively (361 s [177 s;465 s] resp. 278.5 s [150.5 s;395 s]).

During the first 15 min of NR the overall period without active manipulation was longer in the IRS group (median [IQR] 493.5 s [279.25 s;561.5 s]) than in the other two groups (LRS group 203 s [101 s;351 s]; ERS group 335.5 s [237 s;515.75 s]).

### Location of stimulation

**Figure 3:**
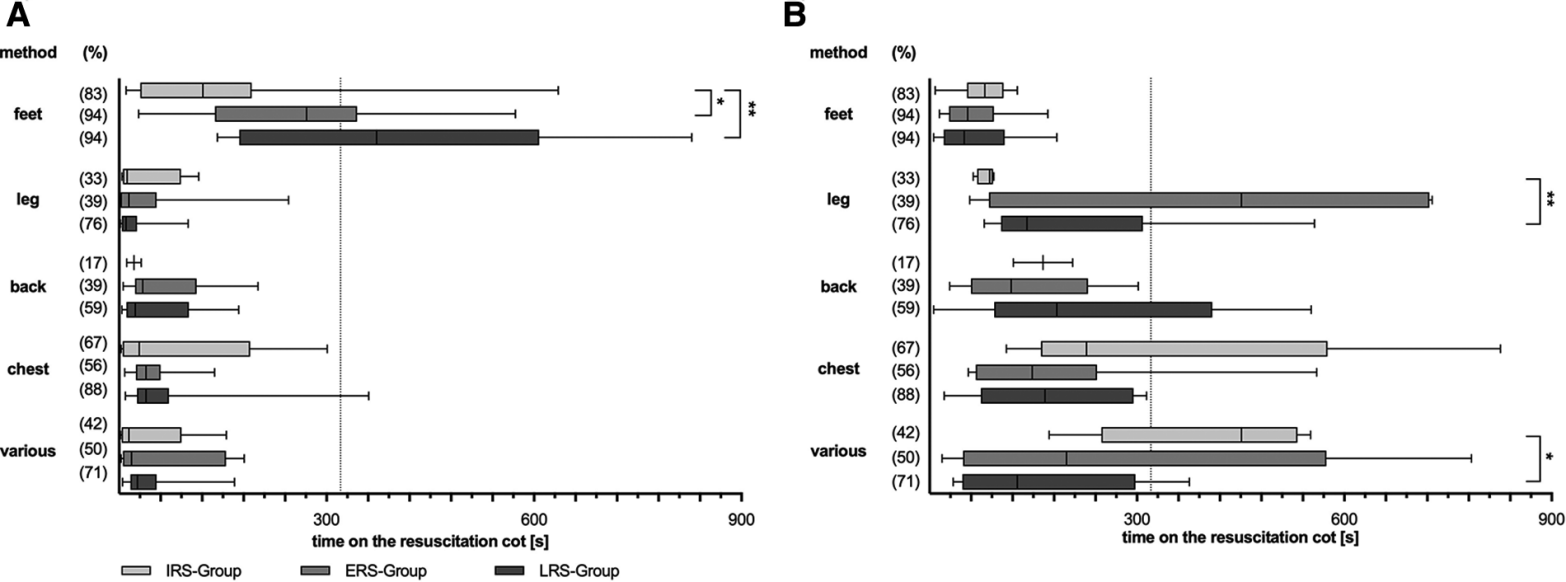
Stimulation procedure divided by different stimulation methods. Overall duration (**A**) and first time (**B**) of different tactile stimulation (TS) methods during the first 15 min of postnatal support. Three subgroups are depicted: LRS, late respiratory stability group; ERS, early respiratory stability group; IRS, immediately respiratory stability group. (%) – proportion of stimulated infants within the particular group; *p*-values – (**A**) **p = 0.005, *p = 0.024; (**B**) **p = 0.009, *p = 0.015. Various stimulation method – defined as stimulation not assignable to one of the other methods.

TS was mainly (75% of infants) initiated at the feet (Figure 3B), whereas the chest was the last region used for stimulation. But 55% of the infants were stimulated at a body region not assignable to the feet, chest, leg or back at least once during the observational period.

The feet were not only the first but also the predominant region for stimulation with an overall duration of feet-stimulation of 210 s [147 s;384 s]. Infants in the LRS group received feet-stimulation for the longest time [372.5 s (173.75 s;607.5 s)], when compared with ERS and IRS group respectively (271 s [138.5 s;344 s] resp. 121 s [30.5 s;191.75 s]) (Figure 3A).

### Concomitant stimulation

**Figure 4:**
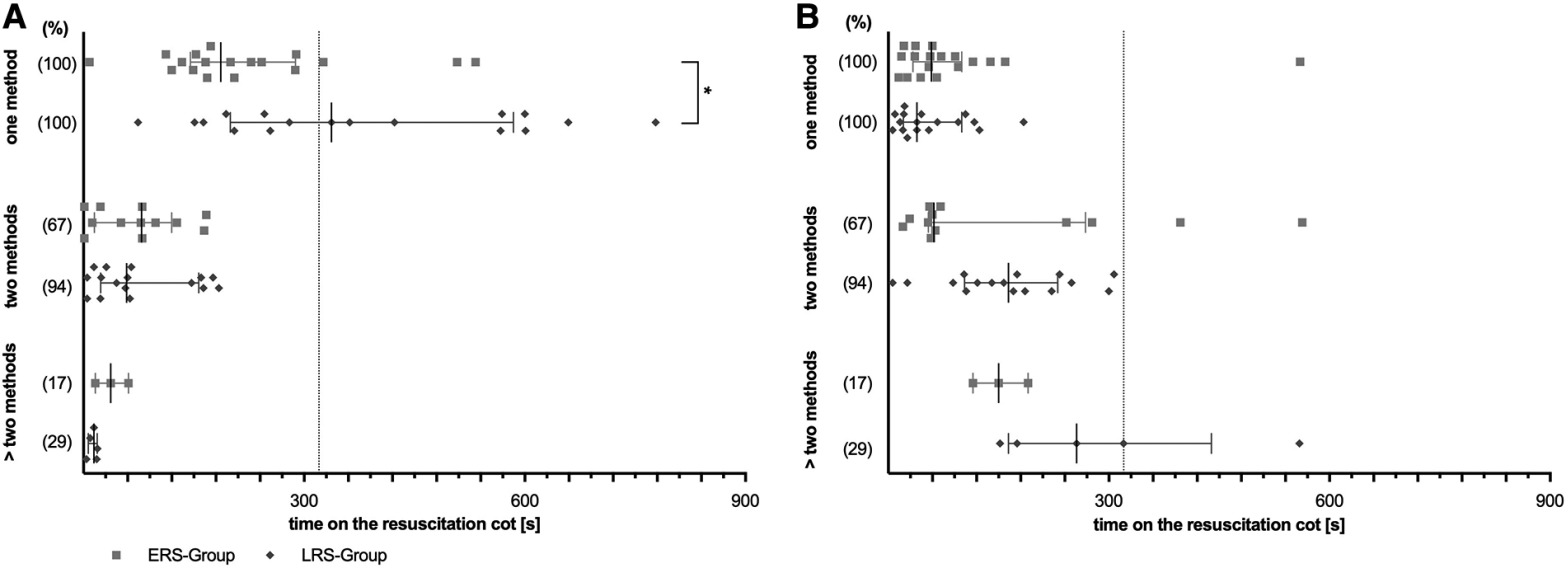
Concomitant stimulations. Overall duration (**A**) and first time (**B**). Scatter dot plot for only one stimulation method up to more than two applicated concomitant stimulation methods. Two subgroups are depicted: LRS, late respiratory stability group; ERS, early respiratory stability group. (%) – proportion of stimulated infants within the particular group; *p*-values - *p = 0.015; *p = 0.015.

Both groups of infants with non-invasive ventilation differed in regards to concomitant stimulation (Figure 4). Infants which achieved respiratory stability early (ERS) received concomitant (two, three or more) stimulations less frequently, but then much earlier and for a longer period of time than infants with late respiratory stability (LRS).

## Discussion

Due to the immaturity of their lungs and central nervous system, preterm infants often require support in postnatal transition (25, 26). Although there is a lack of evidence, current resuscitation guidelines recommend tactile stimulation immediately after birth in apneic infants (4). Furthermore, spontaneous breathing efforts could be increased by changing the arousal state as used in the prevention of apnea of prematurity later on (5). But up until now, there is no agreement regarding the optimal mode, duration or location of TS (12, 13).

By analyzing video recordings of support of postnatal transition of very preterm infants, we were able to show that all infants received TS, regardless of the need for non-invasive ventilation. Furthermore, duration of TS seems to depend on the duration of non-invasive ventilation. Finally, an early onset of concomitant TS seems to be associated with a shorter need for non-invasive ventilation.

Surprisingly and contrary to our hypothesis, we demonstrated that spontaneously breathing preterm infants received TS for a shorter duration and less intense than those infants who needed non-invasive ventilation after birth. The consideration that intense TS may lead to a mask leakage was rebutted recently (16). Contrary, TS during PPV was associated with an increase in respiratory rate (16).

Furthermore, our data suggest that more intense TS, defined as early concomitant TS, could be effective to reduce the need for ventilation. This finding is in line with data from Dekker et al., demonstrating that repetitive, and therefore intense TS is associated with a significant improvement in oxygenation and with a lower FiO_2_ on admission to the NICU compared to infants who only receive standard TS at the discretion of the caregiver (20). These findings could be explained by evidence that TS of a larger surface area and a longer stimulation period may be associated with a greater effect on respiratory stability (16).

In general, all analyzed infants born less than 32^0/7^ weeks of GA were stimulated at least once. A recently published review of the current available data on TS during neonatal resuscitation in the delivery room showed a huge variability in TS of preterm and term infants between 43% and 90% (12). In line with other studies, the preferred stimulation method in our cohort was rubbing and flicking the feet of the infants (14, 20). More than 2/3 of the infants got stimulated at their feet first which may be due to the fact that the NICU-nurse stands at the infants' feet and begins to stimulate the feet after the oxygen sensor is placed routinely. In our institution, the neonatologist who is responsible for respiratory support is usually the second stimulating caregiver which explains the high rate of chest stimulation in more than 30% of the infants after beginning to stimulate the feet.

In line with other investigations, approximately 75% of our very preterm infants needed iPPV or INFL (15, 17). Only about half of those were stimulated within the first minute as recommended in current resuscitation guidelines (4). A delay of TS has been observed in other studies before (14, 15, 18). This may be because postnatal support is often performed after cord clamping in a separate space and that the caregivers initially are focused on initiating mask ventilation, heart rate assessment and the establishing of a stable monitoring.

This study has some limitations. Videos of a single center have been analyzed and no statements can be made about short term outcomes. Due to poor quality of video recordings only a limited number of infants were analyzed. If videography is used for scientific analysis, special focus on the quality of the videos is needed (27), however, in our situation videos were recorded for quality improvement, debriefing and training reasons. At our institution no respiratory function monitor is used and it therefore remains difficult to objectify the effects of TS on breathing efforts. Beside these technical limitations, the study does not allow for any conclusions regarding caregiver's intention of specific interventions.

Despite of the limitations, our data are of great relevance for planning clinical trials on stimulation. Based on our results, we would suggest the following characteristics of TS for the intervention groups of a randomized controlled trial: TS should be initiated early even in very preterm infants who are breathing spontaneously. TS should be more intense in infants who are not breathing sufficient and are in need of non-invasive ventilation. It could be hypothesized that this increase in intensity might be achieved by early concomitant TS of larger body regions. Our data show that TS happened during 9 out of 15 analyzed minutes, showing that “no stimulation” is difficult for a control group and raising the question regarding potential negative stress and limits of effectiveness of that intervention. Nevertheless, we could not assess any obvious short-term side effects.

## Conclusion

This retrospective observational study demonstrated that stimulation of very preterm infants is a commonly used, safe but very subjective method to stimulate spontaneous breathing during postnatal support. The concomitant physical stimulation of different body parts and therefore a larger surface area might be beneficial. A randomized controlled trial is warranted to test optimal mode and duration of TS.

## Data Availability

The raw data supporting the conclusions of this article will be made available by the authors, without undue reservation.
