# Supplementary material for: Tactile stimulation in very preterm infants and their needs of non-invasive respiratory support
Source: Front Pediatr. 2022 Nov 18;10:1041898. doi: 10.3389/fped.2022.1041898 (PMC9715591; doi:10.3389/fped.2022.1041898)
Supplement: Supplementary file 1 [file Table1.docx]

| **supplement table 1: levels of statistical significance** | | | | | | |
| --- | --- | --- | --- | --- | --- | --- |
|  | **IRS^1^**  **(N=12)** | **ERS^1^**  **(N=18)** | **LRS^1^**  **(N=17)** | **p-value** | | |
|  |  |  |  | **IRS/ERS** | **IRS/LRS** | **ERS/LRS** |
| **respiratory support** | | | | | | |
| **iPPV – duration, s** | - | 55 (13.5;95.75) | 155 (79;221) | - | - | *0.004*** |
| **INFL – duration, s** | - | 18 (5.25;31.75) | 20 (5;24.5) | - | - | 0.934 |
| **iPPV – episodes, N** | - | 2 (1.75;4.5) | 4 (3;5.5) | - | - | *0.027** |
| **INFL – attempts, N** | - | 2.5 (0.75;4) | 2 (1;3) | - | - | 0.946 |
| **methods of tactile stimulation** | | | | | | |
| **TS (overall) – duration, s** | 184.5 (52.5;276.75) | 260 (183.25;356.75) | 445 (253;643) | 0.094 | *0.003*** | *0.010** |
| **TS (feet) – duration, s** | 121 (30.5;191.75) | 271 (138.5;344) | 372.5 (173.75;607.5) | *0.024** | *0.005*** | 0.066 |
| **TS (leg) – duration, s** | 11.5 (5.5;89.5) | 14 (2;54) | 10 (4;26) | 1.000 | 0.692 | 0.968 |
| **TS (back) – duration, s** | 21.5 (11;32) | 34 (23;112) | 23.5 (10.5;100.75) | 0.380 | 0.747 | 0.558 |
| **TS (chest) – duration, s** | 29 (5.25;189.5) | 39 (24.25;59.75) | 39 (26;72) | 0.450 | 0.301 | 0.718 |
| **TS (various) – duration, s** | 14 (3.5;90) | 18 (5.5;154.5) | 26.5 (16.5;54.25) | 0.548 | 0.268 | 0.644 |
| **TS (overall) – latency, s** | 83.5 (57;122.5) | 56 (28.25;100) | 39 (20.5;100) | 0.169 | *0.049** | 0.409 |
| **TS (feet) – latency, s** | 80 (54;106.75) | 55 (28;93) | 50 (20.5;108.5) | 0.259 | 0.280 | 0.773 |
| **TS (leg) – latency, s** | 87 (68.75;91.75) | 451 (86;723) | 141 (103.5;308.5) | 0.155 | *0.009*** | 0.452 |
| **TS (back) – latency, s** | 164 (121;207) | 118 (60;229) | 184 (93.5;408.75) | 0.770 | 0.830 | 0.380 |
| **TS (chest) – latency, s** | 227(161.25;575.75) | 148.5 (66.75;242) | 167 (74;295) | 0.110 | 0.107 | 0.912 |
| **TS (various) – latency, s** | 451 (248.5;532) | 198 (48.5;574) | 127 (47.5;297.75) | 0.549 | *0.015** | 0.374 |
| **NAM (overall) – duration, s** | 493.5 (279.25;561.5) | 335.5 (237;515.75) | 203 (101;351) | 0.219 | *0.006*** | 0.060 |
| **NAM (overall) – latency, s** | 102.5 (72.25;118.5) | 278.5 (150.5;395) | 361 (177;465) | *<0.001**** | *<0.001**** | 0.395 |
| **concomitant stimulation** | | | | | | |
| **one method – duration, s** | 148 (46;277.5) | 186.5 (145.25;288.25) | 337 (199.5;584.5) | 0.320 | *0.015** | *0.015** |
| **two methods – duration, s** | 29 (8;36.5) | 79 (14.75;119.75) | 58.5 (23.25;156.75) | 0.140 | 0.117 | 0.981 |
| **> two methods – duration, s** | - | 37 (16;61) | 14 (6.5;18.5) | - | - | 0.101 |
| **one method – latency, s** | 83.5 (57;123.25) | 58.5 (33.5;100) | 39 (20.5;100) | 0.212 | 0.054 | 0.338 |
| **two methods – latency, s** | 192 (131.5;227) | 62 (54.5;268.25) | 163.5 (103.75;230.25) | 0.292 | 0.563 | 0.307 |
| **> two methods – latency, s** | - | 150 (115;190) | 256 (163.5;439.5) | - | - | 0.101 |
| ^1^Depicted values are presented as Median (IQR).  TS - tactile stimulation; iPPV – intermittent positive pressure ventilation; INFL – initial inflations; NAM – no active manipulation  LRS - late respiratory stability: resp. stability > 320s; ERS - early respiratory stability: resp. stability ≤ 320s; IRS - immediate respiratory stability: no need of intermittent positive pressure ventilation or initial inflations | | | | | | |
